# Supplementary material for: Molecular Signature of Small Cell Lung Cancer after Treatment Failure: The MCM Complex as Therapeutic Target
Source: Cancers (Basel). 2021 Mar 10;13(6):1187. doi: 10.3390/cancers13061187 (PMC7998124; doi:10.3390/cancers13061187)
Supplement: Supplementary file 1 [file cancers-13-01187-s001.pdf]

cancers MDPI

Supplementary Materials: Molecular signature of small cell lung cancer after treatment failure: The *MCM* complex as therapeutic target

Shunsuke Misono, Keiko Mizuno, Takayuki Suetsugu, Kengo Tanigawa, Nijiro Nohata, Akifumi Uchida, Hiroki Sanada, Reona Okada, Shogo Moriya, Hiromasa Inoue and Naohiko Seki

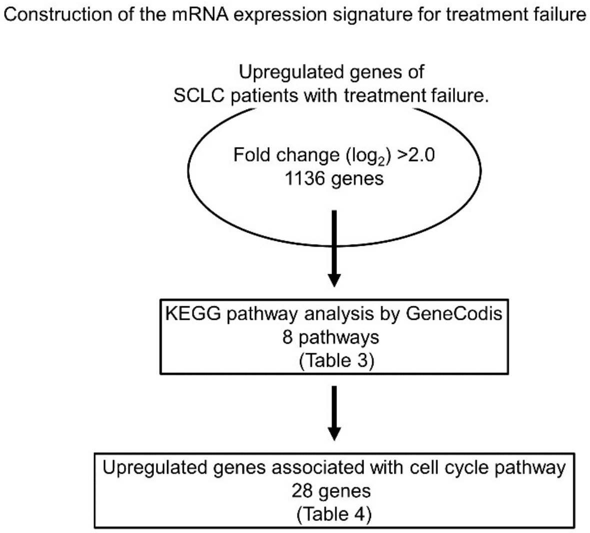

Figure S1. Flowchart of the genes upregulated in SCLC compared with normal tissues classified by KEGG pathway analysis.

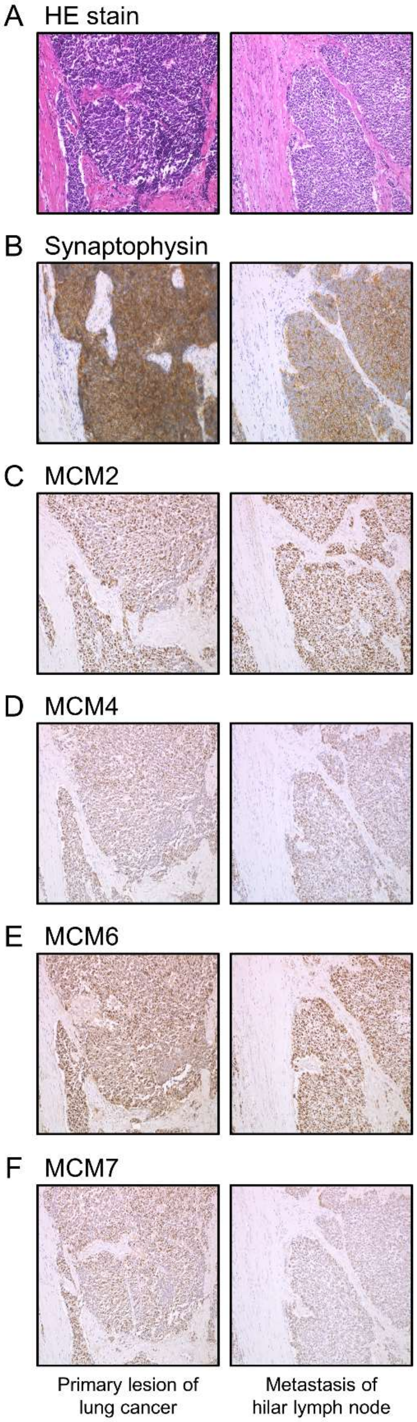

Figure S2. Histopathological findings and immunohistochemical analyses of MCM2, MCM4, MCM6, and MCM7 in case no. 2. (A-F) Overexpression of MCM2, MCM4, MCM6, and MCM7 was observed in the nuclei of cancer cells.

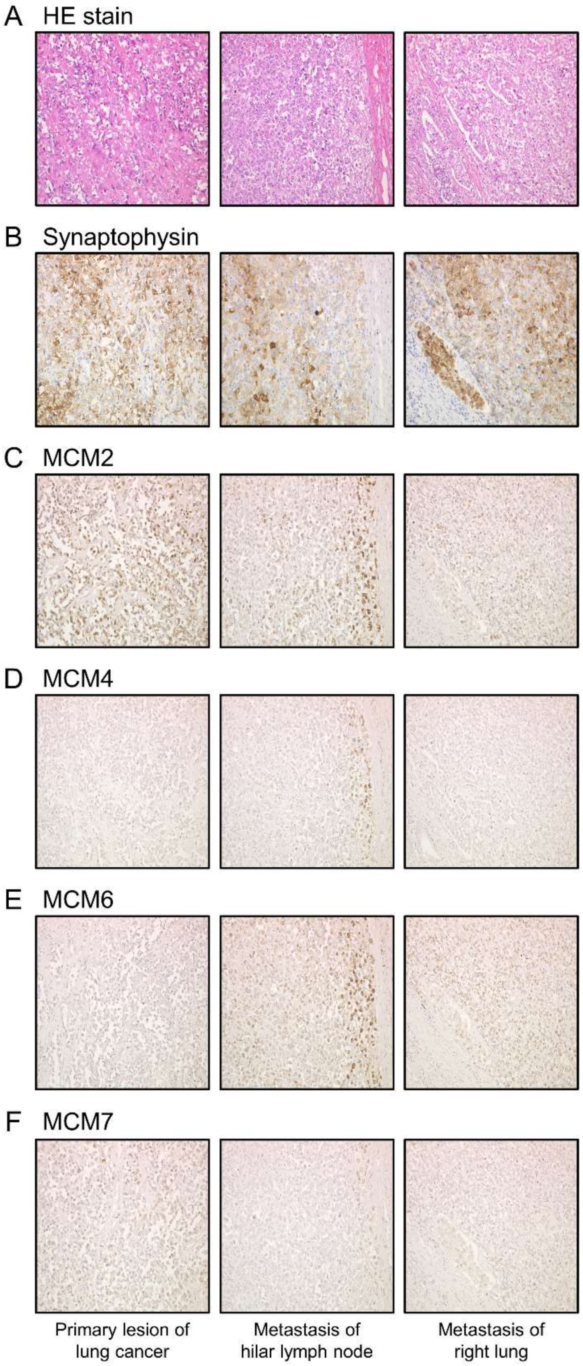

Figure S3. Histopathological findings and immunohistochemical analyses of MCM2, MCM4, MCM6, and MCM7 in case no. 3. (A-F) Overexpression of MCM2, MCM4, MCM6, and MCM7 was observed in the nuclei of cancer cells.

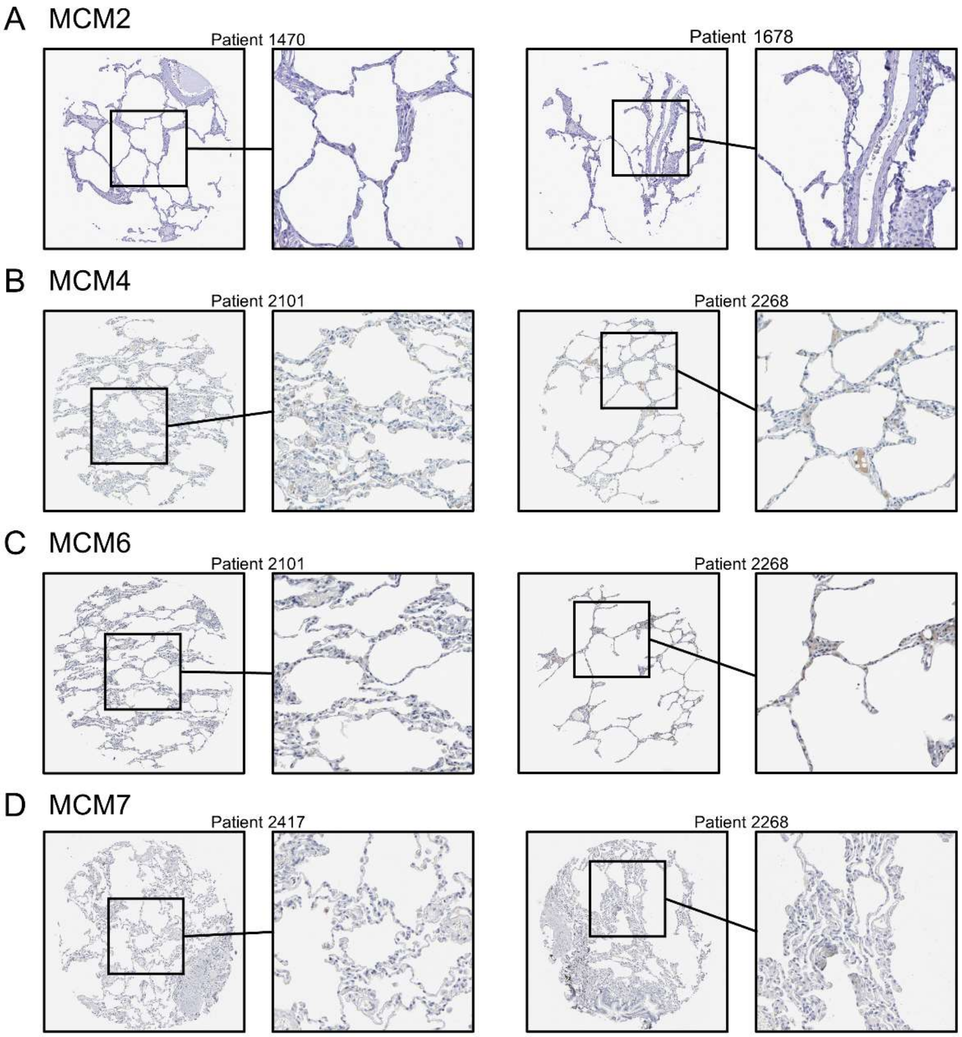

Figure S4. Histopathological findings and immunohistochemical analyses of MCM2, MCM4, MCM6, and MCM7 in normal lung tissue. (A-D) MCM family members were almost not detected in normal lung tissues (Credit: Image from the Human Protein Atlas).

A

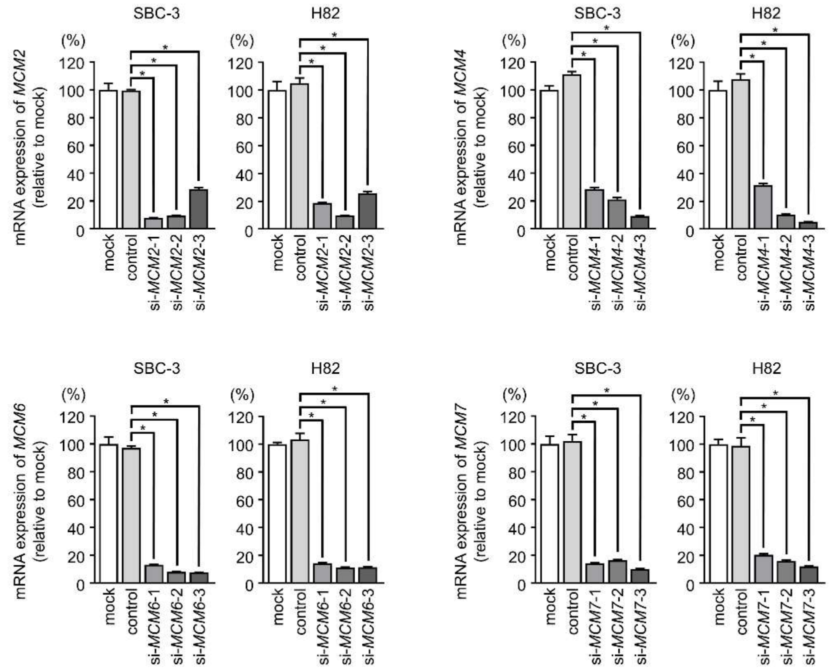
SBC-3
H82
mRNA expression of MCM2 (relative to mock)
mRNA expression of MCM4 (relative to mock)
mRNA expression of MCM6 (relative to mock)
mRNA expression of MCM7 (relative to mock)
(%)
mock
control
si-MCM2-1
si-MCM2-2
si-MCM2-3
si-MCM4-1
si-MCM4-2
si-MCM4-3
si-MCM6-1
si-MCM6-2
si-MCM6-3
si-MCM7-1
si-MCM7-2
si-MCM7-3

B

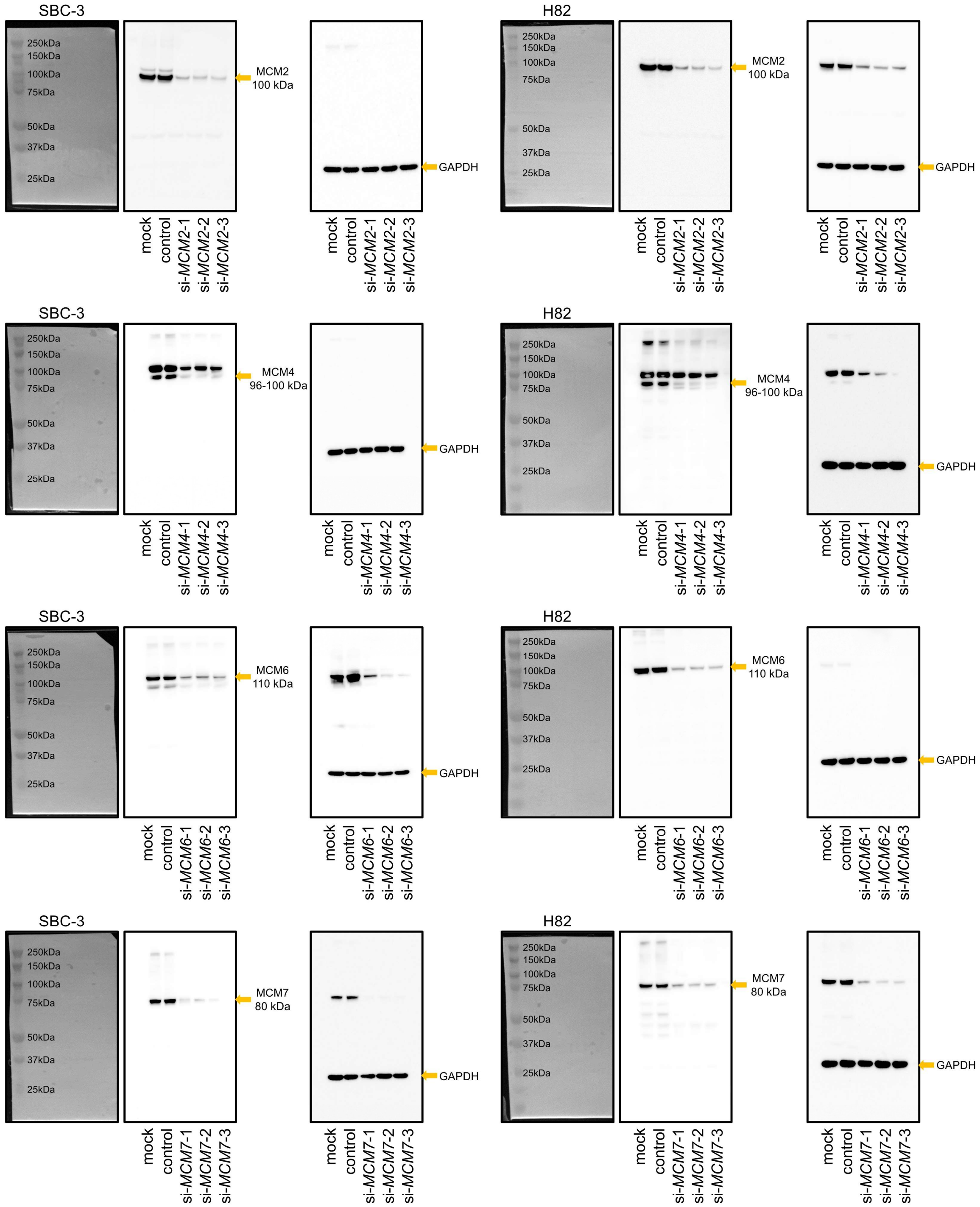
SBC-3
H82
250kDa
150kDa
100kDa
75kDa
50kDa
37kDa
25kDa
MCM2
100 kDa
MCM4
96-100 kDa
MCM6
110 kDa
MCM7
80 kDa
GAPDH
mock
control
si-*MCM2*-1
si-*MCM2*-2
si-*MCM2*-3
si-*MCM4*-1
si-*MCM4*-2
si-*MCM4*-3
si-*MCM6*-1
si-*MCM6*-2
si-*MCM6*-3
si-*MCM7*-1
si-*MCM7*-2
si-*MCM7*-3

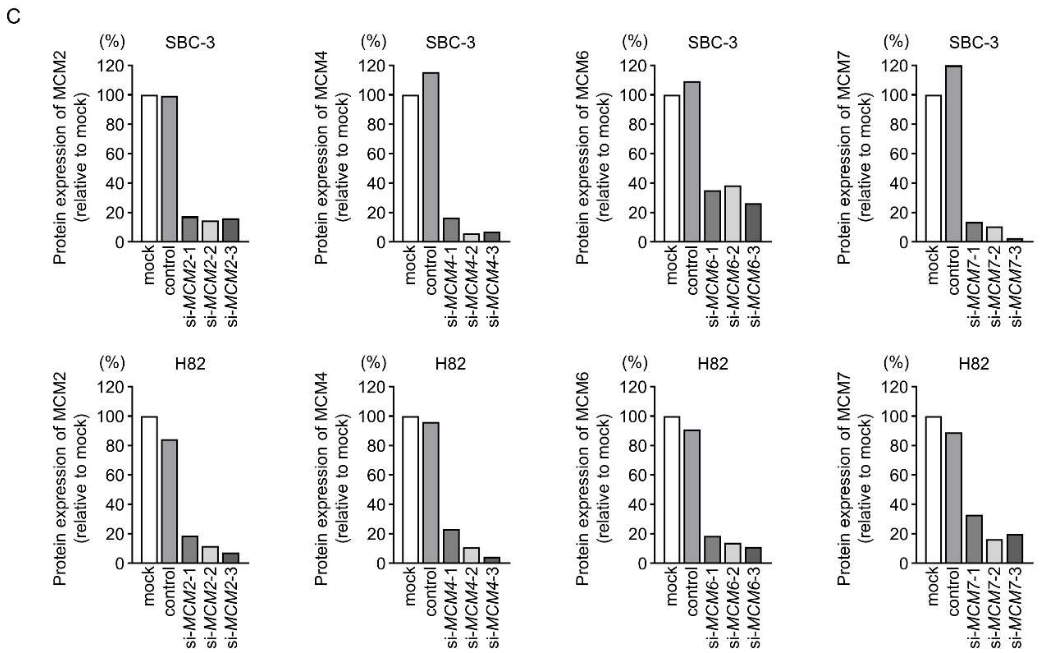

Figure S5. Downregulation of *MCM* family members by siRNA transfection in SCLC cells. Western blots for each MCM protein are shown. *, $p < 0.0001$. (A, B) RT-PCR and western blotting showed that expression levels of both mRNA and protein were markedly reduced by both siRNAs. (C) The western blotting results were quantified by ImageJ.

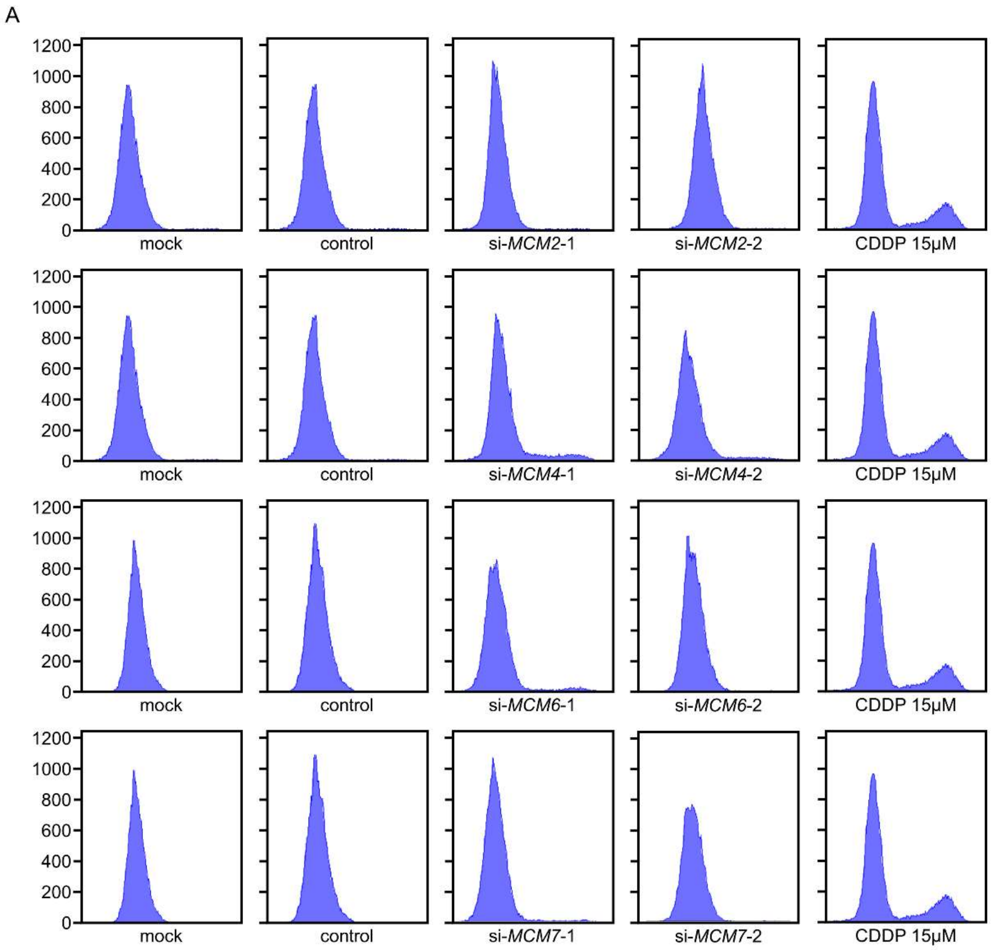
A
1200
1000
800
600
400
200
0
mock
control
si-MCM2-1
si-MCM2-2
CDDP 15μM
si-MCM4-1
si-MCM4-2
si-MCM6-1
si-MCM6-2
si-MCM7-1
si-MCM7-2

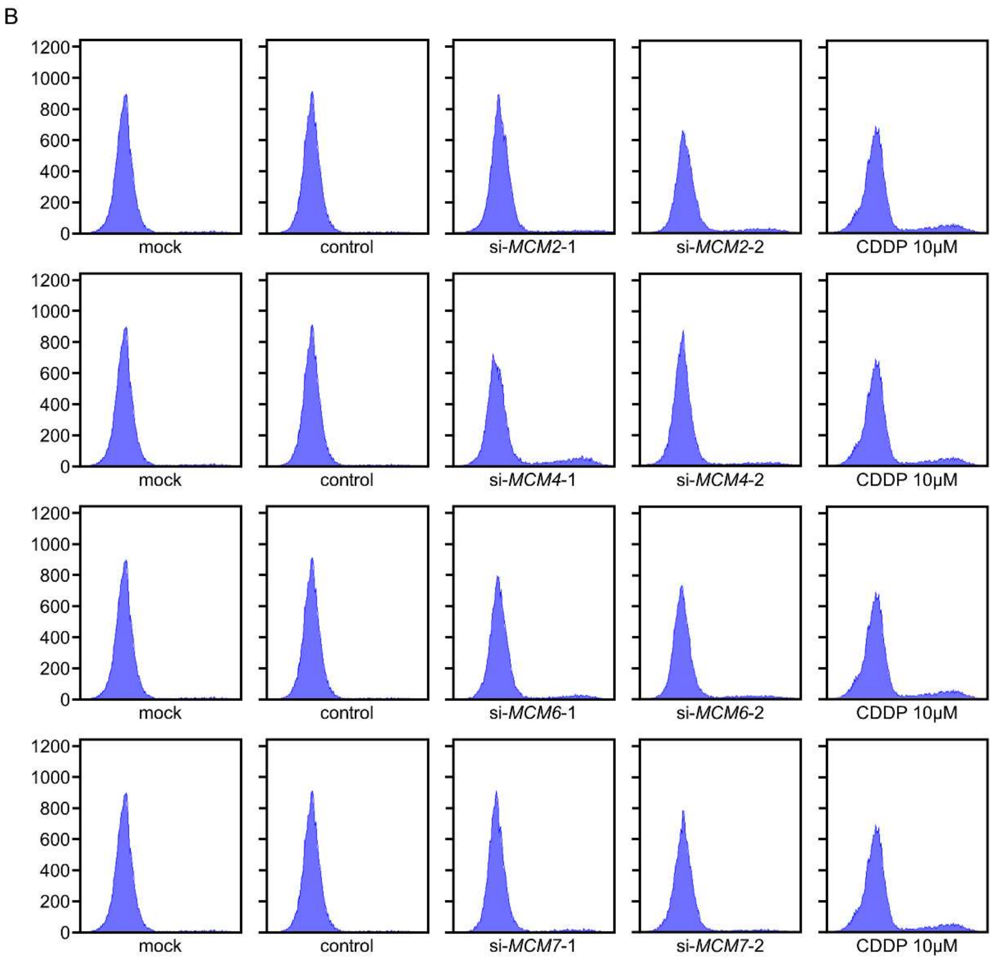

Figure S6. Apoptosis data obtained by flow cytometry from (A) SBC-3 and (B) H82 cells transfected with siRNAs targeting *MCM2*, *MCM4*, *MCM6*, and *MCM7*.

Table S1. Reagent used in this study.

Supplemental Table 1. Reagent used in this study

Antibody	Dilution	Catalog number	Company
Synaptophysin (27G12)	IHC 1:100	413831	Nichirei Biosciences, Inc. (Tokyo, Japan)
Chromogranin	IHC 1:400	412751	Nichirei Biosciences, Inc.
CD56 (1B6)	IHC 1:25	413331	Nichirei Biosciences, Inc.
MCM2	IHC 1:1000<br>WB 1:1000	ab4461	Abcam (Cambridge, UK)
MCM4	IHC 1:1000<br>WB 1:2000	ab4459	Abcam

MCM6	IHC 1:100<br>WB 1:2000	ab4458	Abcam
MCM7	IHC 1:50<br>WB 1:200	ab2360	Abcam
GAPDH	WB 1:20000	MAB374	EMD Millipore (Billerica, MA, USA)

siRNA	Concentration	Catalog number	Company
si-*MCM2*	10 nM	HSS106390, HSS106392, HSS181044	Invitrogen (Carlsbad, CA, USA)
si-*MCM4*	10 nM	HSS106396, HSS106397, HSS106398	Invitrogen
si-*MCM6*	10 nM	HSS106402, HSS106403, HSS106404	Invitrogen
si-*MCM7*	10 nM	HSS106405, HSS106406, HSS181048	Invitrogen

probe	Assay ID	Company
MCM2	Hs01091564_m1	Applied Biosystems (Foster City, CA, USA)
MCM4	Hs00907398_m1	Applied Biosystems
MCM6	Hs00962418_m1	Applied Biosystems
MCM7	Hs00428518_m1	Applied Biosystems
GUSB	Hs00939627_m1	Applied Biosystems

Table S2: Upregulated genes associated with Homologous recombination pathway.

**Entrez GeneID**	**Gene Symbol**	**GeneName**	**Location**	**Log_2 fold change**	**Normalized read count (Log_2)**		***p*-value**
					**Normal lung tissues**	**SCLC tissues**	
8438	*RAD54L*	RAD54-like	1p34.1	3.76	0.06	3.82	0.0078
83990	*BRIP1*	BRCA1 interacting protein C-terminal helicase 1	17q23.2	3.42	0.05	3.47	0.0010
25788	*RAD54B*	RAD54 homolog B	8q22.1	2.84	0.03	2.87	0.0002
672	*BRCA1*	breast cancer 1, early onset	17q21.31	2.46	0.11	2.57	0.0006
5888	*RAD51*	RAD51 recombinase	15q15.1	2.46	0.12	2.58	0.0055
7516	*XRCC2*	X-ray repair complementing defective repair in Chinese hamster cells 2	7q36.1	2.37	0.06	2.42	0.0261
675	*BRCA2*	breast cancer 2, early onset	13q13.1	2.30	-0.07	2.23	0.0005
11073	*TOPBP1*	topoisomerase (DNA) II binding protein 1	3q22.1	2.12	0.05	2.18	0.0003
641	*BLM*	Bloom syndrome, RecQ helicase-like	15q26.1	2.03	0.06	2.10	0.0240

7517	*XRCC3*	X-ray repair complementing defective repair in Chinese hamster cells 3	14q32.33	0.15	0.04	0.20	0.8186

Table S3: Upregulated genes associated with DNA replication pathway.

					Normalized read count (Log_2)		
**Entrez GeneID**	**Gene Symbol**	**GeneName**	**Location**	**Log_2 fold change**	**Normal lung tissues**	**SCLC tissues**	***p*-value**
4173	*MCM4*	minichromosome maintenance complex component 4	8q11.21	3.33	0.20	3.54	0.0004
4171	*MCM2*	minichromosome maintenance complex component 2	3q21.3	2.96	0.01	2.97	0.0008
5427	*POLE2*	polymerase (DNA directed), epsilon 2, accessory subunit	14q21.3	2.69	-0.06	2.62	0.0016
5984	*RFC4*	replication factor C (activator 1) 4	3q27.3	2.31	-0.01	2.30	0.0007
5983	*RFC3*	replication factor C (activator 1) 3	13q13.2	2.26	0.01	2.27	0.0013
4176	*MCM7*	minichromosome maintenance complex component 7	7q22.1	2.20	0.13	2.33	0.0002
10535	*RNASEH2A*	ribonuclease H2, subunit A	19p13.2	2.20	0.08	2.27	0.0020
4175	*MCM6*	minichromosome maintenance complex component 6	2q21.3	2.05	-0.11	1.94	0.0002
5111	*PCNA*	proliferating cell nuclear antigen	20p13	2.04	0.01	2.05	0.0017

Table S4: Upregulated genes associated with p53 signaling pathway.

					Normalized read count (Log_2)		
**Entrez GeneID**	**Gene Symbol**	**GeneName**	**Location**	**Log_2 fold change**	**Normal lung tissues**	**SCLC tissues**	***p*-value**
9134	*CCNE2*	cyclin E2	8q22.1	3.57	0.05	3.62	0.0002

51512	*GTSE1*	G-2 and S-phase expressed 1	22q13.31	3.20	0.04	3.24	0.0011
9133	*CCNB2*	cyclin B2	15q22.2	3.05	0.05	3.10	0.0055
1029	*CDKN2A*	cyclin-dependent kinase inhibitor 2A	9p21.3	3.01	0.24	3.25	0.0047
6241	*RRM2*	ribonucleotide reductase M2	2p25.1	3.00	-0.29	2.71	0.0021
891	*CCNB1*	cyclin B1	5q13.2	2.82	-0.06	2.76	0.0008
898	*CCNE1*	cyclin E1	19q12	2.61	0.06	2.67	0.0041
1111	*CHEK1*	CHK1 checkpoint homolog	11q24.2	2.57	0.00	2.57	0.0088
983	*CDK1*	cyclin-dependent kinase 1	10q21.2	2.40	0.05	2.45	0.0058
56475	*RPRM*	reprimo, TP53 dependent G2 arrest mediator candidate	2q23.3	2.30	0.10	2.40	0.0499
